# Supplementary material for: Device-independent certification of indefinite causal order in the quantum switch
Source: Nat Commun. 2023 Sep 19;14:5811. doi: 10.1038/s41467-023-40162-8 (PMC10509257; doi:10.1038/s41467-023-40162-8)
Supplement: Supplementary file 1 — Supplementary Information [file 41467_2023_40162_MOESM1_ESM.pdf]

Device-independent certification of indefinite causal order in the quantum switch

Supplementary Information

Tein van der Lugt, Jonathan Barrett, Giulio Chiribella

Supplementary Note 1: The quantum switch does not violate causal inequalities

It was shown in [1, 2] that the quantum switch does not violate any causal inequalities in the scenarios previously considered in literature. Here we briefly review this argument, and show why it does not generalise to our scenario involving a constraint on the allowed causal orders arising from the presence of a spacelike-separated party.

We consider the most general correlations $p(a_1 a_2 c|x_1 x_2 z) =: p(\vec{a}c|\vec{x}z)$ observed by parties using just the quantum switch, where Alice 1 and Alice 2 measure the target system inside the two slots of the switch, while Charlie measures the output control and target system. First of all, note that since Charlie chooses his setting z after Alice, this can be written as

$$p(\vec{a}c \mid \vec{x}z) = p(\vec{a} \mid \vec{x}) \cdot p(c \mid \vec{x}z\vec{a}). \tag{1}$$

Alice's marginal correlation $p(\vec{a}|\vec{x})$ is the correlation that would arise if the output control and target systems of the switch were discarded, rather than measured by Charlie. However, discarding the output control qubit on a quantum switch yields the classical switch [3], which is causally separable. Diagrammatically, this can be depicted as [4]

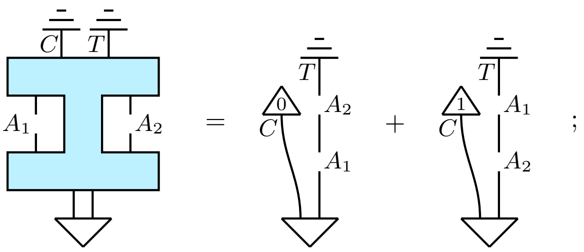

(2)

that is, it is the convex sum (i.e. probabilistic mixture) of two valid processes, both of which correspond to a definite order between the interventions of Alice 1 and 2 (denoted here by gaps in the wires). This directly implies that $p(\vec{a}|\vec{x})$ can be written as a convex sum

$$p(\vec{a} \mid \vec{x}) = \mu p^1(\vec{a} \mid \vec{x}) + (1-\mu) p^2(\vec{a} \mid \vec{x}), \tag{3}$$

where $\mu \in [0,1]$ and where $a_1 \perp\!\!\!\perp_{p^1} x_2$ and $a_2 \perp\!\!\!\perp_{p^2} x_1$; thus, p^1 is compatible with the causal order $A_1 \prec A_2$ and p^2 with the causal order $A_2 \prec A_1$, under the Free Interventions assumption. We can now reintroduce Charlie by using Eq. (1), yielding

$$\begin{aligned} p(\vec{a}c \mid \vec{x}z) &= \mu p^1(\vec{a} \mid \vec{x}) \cdot p(c \mid \vec{x}z\vec{a}) + (1-\mu) p^2(\vec{a} \mid \vec{x}) \cdot p(c \mid \vec{x}z\vec{a}) \\ &=: \mu \tilde{p}^1(\vec{a}c \mid \vec{x}z) + (1-\mu)\tilde{p}^2(\vec{a}c \mid \vec{x}z). \end{aligned} \tag{4}$$

Here, both $\tilde{p}^i$ have no signalling from Charlie to Alice ($\vec{a} \perp\!\!\!\perp_{\tilde{p}^i} z$); therefore $\tilde{p}^1$ is compatible under Free Interventions with the causal order $A_1 \prec A_2 \prec C$ and $\tilde{p}^2$ with $A_2 \prec A_1 \prec C$, proving that the correlation $p(\vec{a}c|\vec{x}z)$ admits an explanation in terms of definite causal orders. For this reason, it does not violate causal inequalities previously studied in literature [1, 2, 5–7].

Turning to the extended scenario studied in this paper, let us consider correlations of the form $p(\vec{a}bc|\vec{x}yz)$ that are generated by the quantum switch entangled to a system in possession of a fourth party, Bob. Analogously to before, we can write

$$p(\vec{a}bc \mid \vec{x}yz) = p(\vec{a}b \mid \vec{x}y) \cdot p(c \mid \vec{x}z\vec{a}), \tag{5}$$

and realise that the entangled switch with discarded output control qubit is causally separable:

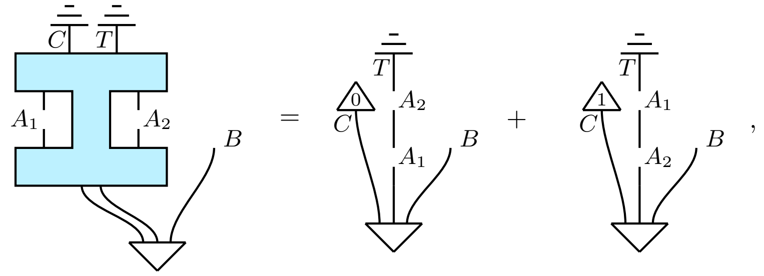

(6)

in such a way that the marginal $p(\vec{a}b|\vec{x}y)$ can be written as a convex sum

$$p(\vec{a}b \mid \vec{x}y) = \mu p^1(\vec{a}b \mid \vec{x}y) + (1-\mu)p^2(\vec{a}b \mid \vec{x}y) \tag{7}$$

where p^1 is compatible with $\{A_1 \prec A_2\} \natural B$ and p^2 with $\{A_2 \prec A_1\} \natural B$. Here $\natural$ denotes the absence of causal relations, as imposed by the Relativistic Causality constraint defined in the main text. More precisely, we have $a_1 b \perp\!\!\!\perp_{p^1} x_2$, $a_2 b \perp\!\!\!\perp_{p^2} x_1$, and $a_1 a_2 \perp\!\!\!\perp_{p^i} y$, $b \perp\!\!\!\perp_{p^i} x_1 x_2$ for $i = 1, 2$.

When we try to reintroduce Charlie's outcome c and write, analogously to Eq. (4),

$$\begin{aligned} p(\vec{a}bc \mid \vec{x}yz) &= \mu p^1(\vec{a}b \mid \vec{x}y) \cdot p(c \mid \vec{x}yz\vec{a}b) + (1-\mu)p^2(\vec{a}b \mid \vec{x}y) \cdot p(c \mid \vec{x}yz\vec{a}b) \\ &=: \mu \tilde{p}^1(\vec{a}bc \mid \vec{x}yz) + (1-\mu)\tilde{p}^2(\vec{a}bc \mid \vec{x}yz), \end{aligned} \tag{8}$$

then we find that in general, the correlations $\tilde{p}^1$ and $\tilde{p}^2$ involve signalling from Bob to Charlie, but (by construction) not from Charlie to Bob. Therefore $\tilde{p}^1$ is compatible with the causal order $A_1 \prec A_2 \prec B \prec C$, and $\tilde{p}^2$ with $A_2 \prec A_1 \prec B \prec C$, so that the total correlation p in principle admits a causal explanation. However, these causal orders are not compatible with the constraint that Bob is causally unrelated to all other parties, which is imposed by the Relativistic Causality assumption when Bob is spacelike-separated. In general, $\tilde{p}^1$ and $\tilde{p}^2$ may exhibit signalling from Bob to Charlie, and they indeed do so for the particular quantum switch correlations considered in the main text. In other words, the decomposition of Eq. (8) does not necessarily allow us to construct a hidden variable model $p(\vec{a}bc\lambda|\vec{x}yz)$ satisfying $p(\,\cdot \mid \cdot\,\lambda) \in \mathcal{DRF}_\lambda$. This leaves open the possibility for violation of inequalities like (6) in the main text.

On the other hand, Eq. (7) tells us that any inequality valid for $\mathcal{DRF}$ in which the outcome c does not appear, such as (*v*)–(*viii*) in Table 1 in the main text, cannot be violated by the quantum switch setup considered here.

Supplementary Note 2: Formalisation of the assumptions

In the main text, we simplified the derivation of the polytope $\mathcal{DRF}$ in Equation (5) by assuming that the only two causal orders allowed by Relativistic Causality are the ones with Hasse diagrams

$$\prec_1 \; := \; \begin{array}{cc} \mathcal{C} & \\ \uparrow & \\ \mathcal{A}_2 & \\ \uparrow & \mathcal{B} \\ \mathcal{A}_1 & \end{array} \qquad \text{and} \qquad \prec_2 \; := \; \begin{array}{cc} \mathcal{C} & \\ \uparrow & \\ \mathcal{A}_1 & \\ \uparrow & \mathcal{B} \\ \mathcal{A}_2 & \end{array} \tag{9}$$

and (in the Free Interventions assumption) that the causal order λ adjudicating between these two possibilities is independent of all setting variables. In fact, however, we wish to take Relativistic Causality to merely constrain the causal order, meaning that it should also allow for causal orders with strictly fewer causal relations between the parties, such as

$$\prec_3 \; := \; \begin{array}{ccc} \mathcal{C} & & \mathcal{A}_2 \\ & \nwarrow \; \nearrow & \\ & \mathcal{A}_1 & \end{array} \quad \mathcal{B} \quad . \tag{10}$$

Moreover, the possibility of these additional causal orders requires us to also consider that of dynamical causal order, wherein the causal order on a subset of parties depends on the setting of

a party in their causal past [2, 7, 8]. (In our case, for example, $\mathcal{A}_1$ might influence which of $\prec_1$ and $\prec_3$ occurs.)

These two simplifications are however justified, for if these additional and potentially dynamical causal orders were included, the resulting correlations would still be members of the polytope $\mathcal{DRF} = \mathrm{conv}(\mathcal{DRF}_1 \cup \mathcal{DRF}_2)$ (and thus would obey the same inequalities as discussed in the main text). The intuitive reason for this is that the additional causal orders allowed by Relativistic Causality (e.g. $\prec_3$) contain strictly fewer causal relations than either of $\prec_1$ and $\prec_2$ (e.g. $\prec_3 \subseteq \prec_1$ as sets). The Free Interventions assumption imposes strictly more no-signalling constraints with respect to such a causal order, yielding correlations that are already in one of $\mathcal{DRF}_1$ and $\mathcal{DRF}_2$. Moreover, by Relativistic Causality no parties are in the causal past of both $\mathcal{A}_1$ and $\mathcal{A}_2$, so their ordering (which is the only aspect in which $\prec_1$ and $\prec_2$ differ) could not be controlled by other parties even if dynamical causal order were allowed.

Here we formalise our three assumptions stated in the main text, generalising them to allow for these additional causal orders as well as for dynamical causal order. We then formalise the paragraph above in Theorem 1

Let λ be a stochastic variable ranging over the set Ω of preorders, i.e. reflexive and transitive relations, on the set of agents $\mathscr{A} := \{\mathcal{A}_1, \mathcal{A}_2, \mathcal{B}, \mathcal{C}\}$. Depending on context, we will also denote λ by $\preceq_\lambda$. For subsets $\mathscr{X}, \mathscr{Y} \subseteq \mathscr{A}$, the condition $\mathscr{X} \npreceq_\lambda \mathscr{Y}$ is understood to mean that $\forall \mathcal{X} \in \mathscr{X}, \mathcal{Y} \in \mathscr{Y} : \mathcal{X} \npreceq_\lambda \mathcal{Y}$ (similarly for expressions such as $\mathcal{X} \npreceq_\lambda \mathscr{Y}$ and $\mathscr{X} \npreceq_\lambda \mathcal{Y}$). Sometimes we will interpret such a condition on λ as an event, i.e. a subset of Ω. Our first two assumptions are on the impossibility of some of the orders in Ω.

Definite Causal Order (DCO). There is a variable λ, ranging over the set of preorders Ω and jointly distributed with the settings and outcomes in a conditional probability distribution $p(\vec{a}bc\lambda|\vec{x}yz)$. It satisfies

$$p(\lambda|\vec{x}yz) = 0 \quad \text{for any } \lambda \in \Omega \text{ that is not antisymmetric.} \tag{11}$$

(That is, the causal order $\preceq_\lambda$ it picks out is always acyclic, i.e. definite.)

Relativistic Causality (RC).

$$p\big(\mathcal{C} \npreceq_\lambda \{\mathcal{A}_1, \mathcal{A}_2\}, \mathcal{B} \npreceq_\lambda \{\mathcal{A}_1, \mathcal{A}_2, \mathcal{C}\}, \{\mathcal{A}_1, \mathcal{A}_2, \mathcal{C}\} \npreceq_\lambda \mathcal{B}\big) = 1. \tag{12}$$

(That is, the causal order $\preceq_\lambda$ satisfies, with certainty, the constraints imposed by the spatiotemporal structure $\prec_g$ discussed in the main text and Figure 1.)

The Free Interventions assumption should be compatible with the existence of dynamical causal orders. We use the following condition, proposed by Oreshkov and Giarmatzi [2]. Here, given a set $\mathscr{X} \subseteq \mathscr{A}$, the equivalence relation $\sim_{\mathscr{X}}$ on Ω is defined by $\lambda \sim_{\mathscr{X}} \mu \iff \lambda \cap (\mathscr{X} \times \mathscr{X}) = \mu \cap (\mathscr{X} \times \mathscr{X})$ and has equivalence classes $[\lambda]_{\mathscr{X}} := \{\mu \in \Omega : \lambda \sim_{\mathscr{X}} \mu\}$.

Free Interventions (FI). For any $\lambda^* \in \Omega$ and parties $\mathcal{A}_1, \ldots, \mathcal{A}_n \in \mathscr{A}$ with settings x_i and outcomes a_i $(i = 1, \ldots, n)$ such that $\{\mathcal{A}_n\} \npreceq_{\lambda^*} \{\mathcal{A}_1, \ldots, \mathcal{A}_{n-1}\}$, the probability

$$p(\lambda \in [\lambda^*]_{\{A_i\}_{i=1}^n}, a_1, \ldots, a_{n-1} \mid x_1, \ldots, x_n) \tag{13}$$

is independent of the setting x_n. (Roughly: given that $\mathcal{A}_n$ does not precede any other $\mathcal{A}_i$, her setting x_n can influence neither the others' outcomes $a_1, \ldots, a_{n-1}$, nor the causal order between them.)

(The term 'causal order' is often meant to refer either to properties of spacetime or to properties of correlations between variables. Here we have instead taken the more general approach that causal order is an a priori relation which is constrained by spacetime via RC (and by DCO) and which constrains correlations via FI. Furthermore, note that FI leads to two types of statistical independences in particular: between settings and hidden variables, and between settings and outcomes conditioned on hidden variables (cf. part (i) and (ii) of the less general assumption stated in the main text). When comparing to discussions of Bell's theorem, these correspond to conditions known as measurement independence (also known as free choice) and parameter

independence, respectively. The assumption that the settings x_1, x_2, y, z of the interventions are freely chosen (i.e. have no causes relevant to other aspects of the experiment) is however central to the justification of both these mathematical conditions. This motivates the name of our third assumption.)

Theorem 1. *For any probability distribution $p(\vec{a}bc\lambda|\vec{x}yz)$ satisfying Definite Causal Order, Relativistic Causality, and Free Interventions as defined above, the observed marginal distribution $p(\vec{a}bc|\vec{x}yz)$ is in $\mathcal{DRF}$ (defined in Eq. (5) of the main text).*

Proof. Denote by $\min_\lambda$ the set of $\preceq_\lambda$-minimal elements of $\mathscr{A} := \{\mathcal{A}_1, \mathcal{A}_2, \mathcal{B}, \mathcal{C}\}$ and by $(\mathcal{A}_1 \in \min_\lambda) \subseteq \Omega$ the event $\{\lambda \in \Omega : \mathcal{A}_1 \in \min_\lambda\}$. $\perp\!\!\!\perp$ denotes independence conditioned on settings: for example, for $S \subseteq \Omega$, $a_1 b \perp\!\!\!\perp x_2 \mid S$ is short for

$$p(a_1 b \mid x_1 x_2 y z, \lambda \in S) = p(a_1 b \mid x_1 x_2' y z, \lambda \in S) \quad \text{for all } a_1, b, x_1, x_2, x_2', y, z \tag{14}$$

while $a_1 b \perp\!\!\!\perp x_2$ means $a_1 b \perp\!\!\!\perp x_2 \mid \Omega$.

Lemma 1. *DCO and FI imply that $(\mathcal{A}_1 \in \min_\lambda) \perp\!\!\!\perp \vec{x}yz$.*

Proof. For $\mathscr{X} \subseteq \mathscr{A}$, write $(\min_\lambda = \mathscr{X})$ for the event $\{\lambda : \min_\lambda = \mathscr{X}\} \subseteq \Omega$. In Ref. [2], this is notated as $[\mathscr{X}]^{\mathrm{I}}$. Their Proposition 2.3 (whose proof relies on DCO) implies that $p(\min_\lambda = \mathscr{X}|\vec{x}yz)$ is independent of $\vec{x}yz$. Since $(\mathcal{A}_1 \in \min_\lambda) = \bigcup_{\mathcal{A}_1 \in \mathscr{X} \subseteq \mathscr{A}} (\min_\lambda = \mathscr{X})$ and this union is disjoint, $p(\mathcal{A}_1 \in \min_\lambda \mid \vec{x}yz)$ is also independent of $\vec{x}yz$. □

Thus $p(\vec{a}bc|\vec{x}yz)$ is a convex mixture of $p(\vec{a}bc|\vec{x}yz, \mathcal{A}_1 \in \min_\lambda)$ and $p(\vec{a}bc|\vec{x}yz, \mathcal{A}_1 \notin \min_\lambda)$ (assume, without loss of generality, that $0 < p(\mathcal{A}_1 \in \min_\lambda) < 1$). The Lemma below shows that the former is in $\mathcal{DRF}_1$ while the latter is in $\mathcal{DRF}_2$, thereby completing the proof of the Theorem.

Lemma 2. *DCO, RC and FI imply the following conditional independences.*

(i) $a_1 a_2 b \perp\!\!\!\perp z \mid \mathcal{A}_1 \in \min_\lambda$ *and* $a_1 a_2 b \perp\!\!\!\perp z \mid \mathcal{A}_1 \notin \min_\lambda$*;*
(ii) $a_1 a_2 c \perp\!\!\!\perp y \mid \mathcal{A}_1 \in \min_\lambda$ *and* $a_1 a_2 c \perp\!\!\!\perp y \mid \mathcal{A}_1 \notin \min_\lambda$*;*
(iii) $b \perp\!\!\!\perp x_1 x_2 z \mid \mathcal{A}_1 \in \min_\lambda$ *and* $b \perp\!\!\!\perp x_1 x_2 z \mid \mathcal{A}_1 \notin \min_\lambda$*;*
(iv) $a_1 b \perp\!\!\!\perp x_2 \mid \mathcal{A}_1 \in \min_\lambda$*;*
(v) $a_2 b \perp\!\!\!\perp x_1 \mid \mathcal{A}_1 \notin \min_\lambda$*.*

Proof. *(i)* Let $S \subseteq \Omega$ be $(\mathcal{A}_1 \in \min_\lambda)$ or $(\mathcal{A}_1 \notin \min_\lambda)$. By RC, $p(\mathcal{C} \not\preceq_\lambda \{\mathcal{A}_1, \mathcal{A}_2, \mathcal{B}\}) = 1$ (Eq. (12)). Therefore

$$p(\vec{a}b, \lambda \in S \mid \vec{x}yz) = \sum_{\lambda^* \in S \text{ and } \mathcal{C} \not\preceq_{\lambda^*} \{\mathcal{A}_1, \mathcal{A}_2, \mathcal{B}\}} p(\vec{a}b, \lambda = \lambda^* \mid \vec{x}yz), \tag{15}$$

each term of which is independent of z by FI (Eq. (13)).

(ii) Similar to *(i)*, using $p(\mathcal{B} \not\preceq_\lambda \{\mathcal{A}_1, \mathcal{A}_2, \mathcal{C}\}) = 1$.

(iii) Let S again be $(\mathcal{A}_1 \in \min_\lambda)$ or $(\mathcal{A}_1 \notin \min_\lambda)$. In both cases, S can be written as a disjoint union $\bigcup_i (\min_\lambda = \mathscr{X}_i)$, where all $\mathscr{X}_i \subseteq \mathscr{A}$. By RC, $p(\mathcal{B} \in \min_\lambda) = 1$. Therefore

$$\begin{aligned} p(\lambda \in S, b \mid \vec{x}yz) &= p(\lambda \in S, \mathcal{B} \in \min_\lambda, b \mid \vec{x}yz) \\ &= \sum_{i : \mathcal{B} \in \mathscr{X}_i} p(\min_\lambda = \mathscr{X}_i, b \mid \vec{x}yz), \end{aligned} \tag{16}$$

which by Proposition 2.3 of Ref. [2] is independent of x_1, x_2, and z.

(iv) Similar to *(iii)*, with $S = (\mathcal{A}_1 \in \min_\lambda)$ and b replaced by $a_1 b$.

(v) By DCO, we can assume without loss of generality that all $\lambda \in \Omega$ are antisymmetric. Then the conditions of RC imply that necessarily one of $\mathcal{A}_1$ and $\mathcal{A}_2$ has no other party in their causal past: i.e. $p(\mathcal{A}_2 \in \min_\lambda \mid \vec{x}yz, \mathcal{A}_1 \notin \min_\lambda) = 1$. Therefore

$$\begin{aligned} p(a_2 b, \mathcal{A}_1 \notin \min_\lambda \mid \vec{x}yz) &= p(a_2 b, \mathcal{A}_1 \notin \min_\lambda, \mathcal{A}_2 \in \min_\lambda \mid \vec{x}yz) \\ &= \sum_{\mathscr{X} \subseteq \mathscr{A} : \mathcal{A}_1 \notin \mathscr{X}, \mathcal{A}_2 \in \mathscr{X}} p(a_2 b, \min_\lambda = \mathscr{X} \mid \vec{x}yz) \end{aligned} \tag{17}$$

which by Proposition 2.3 of [2] is independent of x_1. □□

Supplementary Note 3: Proof of Inequality (*iv*) in Table 1

Recall Inequality (iv):

$$\begin{aligned}1/2\big[&p(a_1=0\mid x_1x_2=10)+p(a_2=0\mid x_1x_2=01)-p(a_1a_2=00\mid x_1x_2=11)\big]\\ &p(b=0,a2=x1|x2y=00)+p((x_2a_1+(x_2\oplus 1)c)\oplus b=x_2y\mid x_1=x_2)\leq 7/4.\end{aligned} \tag{18}$$

Denote by β the first three terms of the inequality, without the factor 1/2:

$$\beta := p(a_1=0\mid 10)+p(a_2=0\mid 01)-p(a_1a_2=00\mid 11). \tag{19}$$

Here the unlabelled conditional variables denote x_1 and x_2, respectively. Similarly to the proof of Theorem 1 in Methods, we prove the inequality for $\mathcal{DRF}_1$ and $\mathcal{DRF}_2$ separately. In both polytopes, the proof proceeds by first bounding the outcome probabilities for a_1 and then using a monogamy inequality. For $p\in\mathcal{DRF}_1$, we have $a_1 \perp\!\!\!\perp_p x_2$ so that

$$p(a_1=0\mid 11)=p(a_1=0\mid 10)\geq\beta-1, \tag{20}$$

while for $p\in\mathcal{DRF}_2$, $a_2\perp\!\!\!\perp_p x_1$ implies

$$\begin{aligned}p(a_1=1\mid 11)&\geq p(a_1a_2=10\mid 11)\\ &=p(a_2=0\mid 11)-p(a_1a_2=00\mid 11)\\ &=p(a_2=0\mid 01)-p(a_1a_2=00\mid 11)\geq\beta-1.\end{aligned} \tag{21}$$

Applying the monogamy inequality of Ref. [9], now to the CHSH-like term of Inequality (iv), yields the following bound for $p\in\mathcal{DRF}_i$, where $i=1,2$:

$$\begin{aligned}p((x_2a_1&+(x_2\oplus 1)c)\oplus b=x_2y\mid x_1=x_2)\\ &\leq\frac{5}{4}-\frac{1}{2}p(a_1=i-1\mid 11)\\ &\leq\frac{5}{4}-\frac{1}{2}(\beta-1)=\frac{7}{4}-\frac{1}{2}\beta.\end{aligned} \tag{22}$$

Combining this with Equation (19) completes the proof. □

References

1. Araújo, M. *et al.* Witnessing Causal Nonseparability. *New Journal of Physics* **17,** 102001 (2015).
2. Oreshkov, O. & Giarmatzi, C. Causal and Causally Separable Processes. *New Journal of Physics* **18,** 093020 (2016).
3. Chiribella, G., D'Ariano, G. M., Perinotti, P. & Valiron, B. Quantum Computations without Definite Causal Structure. *Physical Review A* **88,** 022318 (2013).
4. Coecke, B. & Kissinger, A. *Picturing Quantum Processes: A First Course in Quantum Theory and Diagrammatic Reasoning* 1st ed. (Cambridge University Press, 2017).
5. Oreshkov, O., Costa, F. & Brukner, Č. Quantum Correlations with No Causal Order. *Nature Communications* **3,** 1092 (2012).
6. Branciard, C., Araújo, M., Feix, A., Costa, F. & Brukner, Č. The Simplest Causal Inequalities and Their Violation. *New Journal of Physics* **18,** 013008 (2015).
7. Abbott, A. A., Giarmatzi, C., Costa, F. & Branciard, C. Multipartite Causal Correlations: Polytopes and Inequalities. *Physical Review A* **94,** 032131 (2016).
8. Gogioso, S. & Pinzani, N. *The Geometry of Causality* `http://arxiv.org/abs/2303.09017` (2023). preprint.
9. Barrett, J., Kent, A. & Pironio, S. Maximally Nonlocal and Monogamous Quantum Correlations. *Physical Review Letters* **97,** 170409 (2006).
